# Infantile atopic dermatitis – increasing severity predicts negative impacts on maternal and infant sleep: a mixed methods study

**DOI:** 10.1186/s13223-024-00883-x

**Published:** 2024-03-22

**Authors:** Zoe Harbottle, Amanda Nötzel, Michael A. Golding, Manvir Bhamra, Isac Kopsch, Erik Wilking, Marina Jonsson, Elissa M. Abrams, Michelle A. Halbrich, Elinor Simons, Leslie E. Roos, Jill A. Keddy-Grant, Thomas V. Gerstner, Jo-Anne St-Vincent, Sandra Ekström, Jennifer L. P. Protudjer

**Affiliations:** 1 Department of Pediatrics and Child Health, Rady Faculty of Health Sciences, Max Rady College of Medicine, University of Manitoba, Winnipeg, MB Canada; 2 Children’s Hospital Research Institute of Manitoba, 501G-715 McDermot Avenue, Winnipeg, MB Canada; 3 Department of Food and Human Nutritional Sciences, Faculty of Agricultural and Food Sciences, University of Manitoba, Winnipeg, MB Canada; 4 Karolinska Institutet, Stockholm, Sweden; 5 Institute of Environmental Medicine, Karolinska Institutet, Stockholm, Sweden; 6 Centre for Occupational and Environmental Medicine, Stockholm County Council, Stockholm, Sweden; 7 Department of Clinical Science and Education, Södersjukhuset, Karolinska Institutet, Stockholm, Sweden; 8 Department of Pediatrics and Child Health, Section of Allergy and Clinical Immunology, Rady Faculty of Health Sciences, Max Rady College of Medicine, University of Manitoba, Winnipeg, MB Canada; 9 Division of Allergy and Immunology, Department of Pediatrics, Faculty of Medicine, University of British Columbia, Vancouver, BC Canada; 10 Children’s Allergy & Asthma Education Centre, Health Sciences Centre Winnipeg, Winnipeg, MB Canada; 11 Department of Psychology, Faculty of Arts, University of Manitoba, Winnipeg, MB Canada; 12 Department of Pediatrics and Child Health, Section of Dermatology, Rady Faculty of Health Sciences, Max Rady College of Medicine, University of Manitoba, Winnipeg, MB Canada; 13 George and Fay Yee Centre for Healthcare Innovation, Winnipeg, MB Canada

**Keywords:** Atopic dermatitis, Infant sleep, Maternal sleep, Mixed methods, Sleep disturbance

## Abstract

**Background:**

While the impacts of atopic dermatitis (AD) on maternal and child sleep outcomes have been previously explored, less is known about the associations between infantile AD and sleep quality and quantity.

**Objective:**

To describe the perceived causes of AD-associated maternal sleep disturbances and the association between AD severity and infant sleep outcomes.

**Methods:**

Mothers with infants aged < 19 months old with a diagnosis of AD were recruited from social media and medical clinics in Winnipeg, Canada between October 2021 and May 2022. Infant AD severity was classified using maternal-reported data on the Patient-Oriented Scoring Atopic Dermatitis tool (PO-SCORAD). Quantitative data were collected via a series of questionnaires with a subset of mothers subsequently completing semi-structured interviews. Quantitative and qualitative data were integrated in the discussion.

**Results:**

Mothers of infants with moderate/severe AD (6/12) were more likely to report their infant suffering from a higher degree of sleeplessness (i.e., ≥ 5 on a scale of 0–10) over the past 48 h compared to mothers of infants with mild AD (0/18). This was supported by qualitative findings where mothers described how their infant’s sleep quality and quantity worsened with AD severity. Additionally, 7/32 mothers reported that their child’s AD, regardless of severity, disturbed their sleep. Maternal sleep loss was most commonly attributed to infant itching (6/7), followed by worry (4/7).

**Conclusion:**

Infantile AD severity was associated with worse sleep outcomes for both mothers and infants. We propose that maternal and infantile sleep quality and quantity can be improved by reducing AD severity through adherence to topical treatments.

**Supplementary Information:**

The online version contains supplementary material available at 10.1186/s13223-024-00883-x.

## Introduction

Up to 15% of infants are affected by atopic dermatitis (AD), a chronic skin disease, with a relatively high rate of relapse [1]. AD is characterized by dry skin, pruritus, and rashes, which, in infancy, typically present on the face and extensor aspects of the arms and legs [1]. The severity and manifestation of AD can vary considerably across patients and seasons. Diagnosis can occur at any point in life, however, AD is most common among infants, with the highest incidence rate being between 3 and 6 months old [2].

The impact of AD on maternal and child sleep quality and quantity has been characterized in a small number of studies, which have demonstrated consistently negative outcomes including difficulties falling asleep, increased nocturnal wakening, and involuntary early morning awakening [5–7]. However, gaps in the literature remain as previous studies have typically focused solely on maternal sleep outcomes, without consideration of the causes of sleep disturbances, or have only indirectly examined child outcomes [5, 6]. Research in this area remains limited, formed primarily of quantitative studies with a lack of qualitative studies. Moreover, the previous literature has rarely focused on how AD impacts sleep quality and quantity in infants.

Therefore, in the present study, we aimed to address the gaps within previous research by utilizing a mixed-methods approach to characterize the perceived causes of maternal sleep disturbances, and the association between AD severity and sleep within the infantile population.

## Methods

### Study design and participant recruitment

This mixed-methods study followed a sequential explanatory design with data integration in the discussion. Mothers aged 18 + years with an infant aged < 19 months old with a diagnosis of AD and who were comfortable reading and speaking English were recruited from social media as well as allergy and dermatology clinics in Winnipeg, Manitoba, Canada between October 2021 and May 2022. Upon providing informed consent, mothers completed a series of self-report questionnaires to collect quantitative data. Those specific to the present study are described below. A subset of mothers, who agreed to be contacted for further research related to the overarching AD project, also completed semi-structured interviews following the quantitative questionnaires. Qualitative interview questions were developed *a priori* by content experts and refined subsequent to quantitative data analysis and as the series of interviews progressed.

### Quantitative measures

Mothers were asked to complete several questionnaires that asked about their household’s socio-demographics, medical history, and their infant’s AD severity and sleep quality.

### Primary outcomes

The primary outcomes were sleep quality and quantity. Data on sleep was collected from different questionnaires: an ad-hoc questionnaire focused on the infant’s AD history and management, the Perinatal Anxiety Screening Scale (PASS) and the Patient-Oriented Scoring of Atopic Dermatitis (PO-SCORAD) tool. In completing the AD questionnaire, participants were asked several questions pertaining to sleep quality: “Does your baby’s atopic dermatitis keep *you* awake at night?” “Does your baby’s AD keep *him/her* awake at night?”, “Does your baby receive any medicine to help him/her sleep?”. Participants had the option to answer these questions “yes” or “no”. If a participant indicated that their infant’s AD did keep them up at night, they were also asked the cause (i.e., itching, need to treat, stress, worry, or other). Questions from the PASS used in this study included “How often, over the past month, have you experienced being upset about repeated memories, dreams, or nightmares?” which assessed maternal sleep quality and “How often, over the past month, have you experienced difficulty sleeping even when you have the chance to sleep?” which assessed maternal sleep quantity. Possible answers were; not at all; sometimes; often; or almost always. These possible answers were recoded into no (“not at all”) or yes (“sometimes; often or almost always”). In addition to items described above, infant sleep quality was also evaluated using the PO-SCORAD tool, where infant sleeplessness was assessed on a scale from 0 to 10, where 0 was no sleeplessness and 10 was the worst imaginable sleeplessness.

### Sociodemographics

Mothers were asked to report socio-demographic data for themselves and their infant. Maternal characteristics included: mothers’ age, seasonality, ethnicity, education, and annual household income.

Mother-reported infant characteristics included age (in months), sex (male or female), and allergic comorbidities (asthma, rhinitis, and/or food allergy; not mutually exclusive). AD treatment was reported based on yes or no questions regarding: if moisturizers were used on the infant’s skin, if medications had ever been used on the infant’s skin (e.g., hydrocortisone, betamethasone, etc.), and if other medications had been used for the infant’s AD, such as antibiotics.

### AD severity

AD severity was defined using the PO-SCORAD tool which assesses different areas of the skin to determine the extent of AD on the whole body. Due to the COVID-19 restrictions, the assessment had to be done by the infants’ parents. In completing the measure, participants rated the intensity of their infant’s AD symptoms (i.e., redness, swelling, oozing/crusting, scratch marks, skin thickening, and dryness) on a 4-point scale (i.e., none = 0; mild = 1; moderate = 2; severe = 3) [8]. The subjective symptoms, itch, and sleeplessness, were evaluated on a scale from 0 to 10, where 0 was no itch/sleeplessness and 10 was the worst imaginable itch/sleeplessness [8]. The minimum and maximum PO-SCORAD scores are 0 and 103. A total PO-SCORAD score of < 25 was considered as mild AD, 25–50 moderate and a PO-SCORAD > 50 was considered severe AD [9]. Due to a low number of moderate and severe cases each, these two groups were combined in this study.

### Quantitative analyses

Descriptive statistics were used to describe the demographic characteristics of the studied sample and to describe mothers’ sleep quality and quantity and infant sleep. For all demographic variables, missing data was excluded. Information from PO-SCORAD on infant sleeplessness was used to compare cases of mild AD to cases with moderate/severe AD. Binary logistic regression was used to determine the association between AD severity and maternal sleep quality as well as quantity in one crude and one adjusted model.

Potential confounders were identified through a directed acyclic graph (DAG). The DAG resulted in the minimal sufficient adjustment sets of: AD treatment, infant age, infant allergic comorbidity, maternal ethnicity, maternal education, and seasonality. To not over-adjust for socioeconomic status (SES) [10], only maternal education was included in the regression analysis (Additional file 1). Owing to missing observations for seasonality, infant allergic comorbidities, and AD treatment, the model was not estimable, and we had to limit the regression model to the following potential confounders in the adjusted model: maternal ethnicity, maternal education, and infant age (Additional file 1).

Odds ratios (OR) with 95% confidence intervals (CI), were estimated. A *p*-value, < 0.05, was determined *a priori* as statistically significant. Data analysis was performed using Stata Version 17.0 (College Station, Texas).

### Qualitative data collection

Subsequent to quantitative data collection, a self-selected subset of mothers with infants with AD completed qualitative semi-structured interviews. All mothers who were asked to participate in the qualitative arm of the study had agreed to be contacted for future research related to the overarching AD project. A total of 20 mothers were contacted, 13 provided consent, and 10 completed an interview. All interviews were conducted over the phone by trained research assistants who followed a semi-structured guide (Table 1). While this guide provided the foundational questions for the interview, the interviewers were afforded some flexibility to modify the questions to the individual’s circumstances and in relation to the overall quantitative findings. Each qualitative interview was audio-recorded and transcribed verbatim. Transcripts were then analyzed by two research assistants, who worked independently, but concurrently, using thematic analysis (ZH, MB). Both analysts were supervised by two authors (MG, JP) who possess extensive knowledge of qualitative methods [11].

**Table 1:** Select questions from the interview guide

Please describe both your baby’s and your sleeping patterns.
How has your baby’s atopic dermatitis affected your sleep?
How has caring for your infant with atopic dermatitis impacted your levels of stress or mood?

### Qualitative analyses

**Fig. 1:**
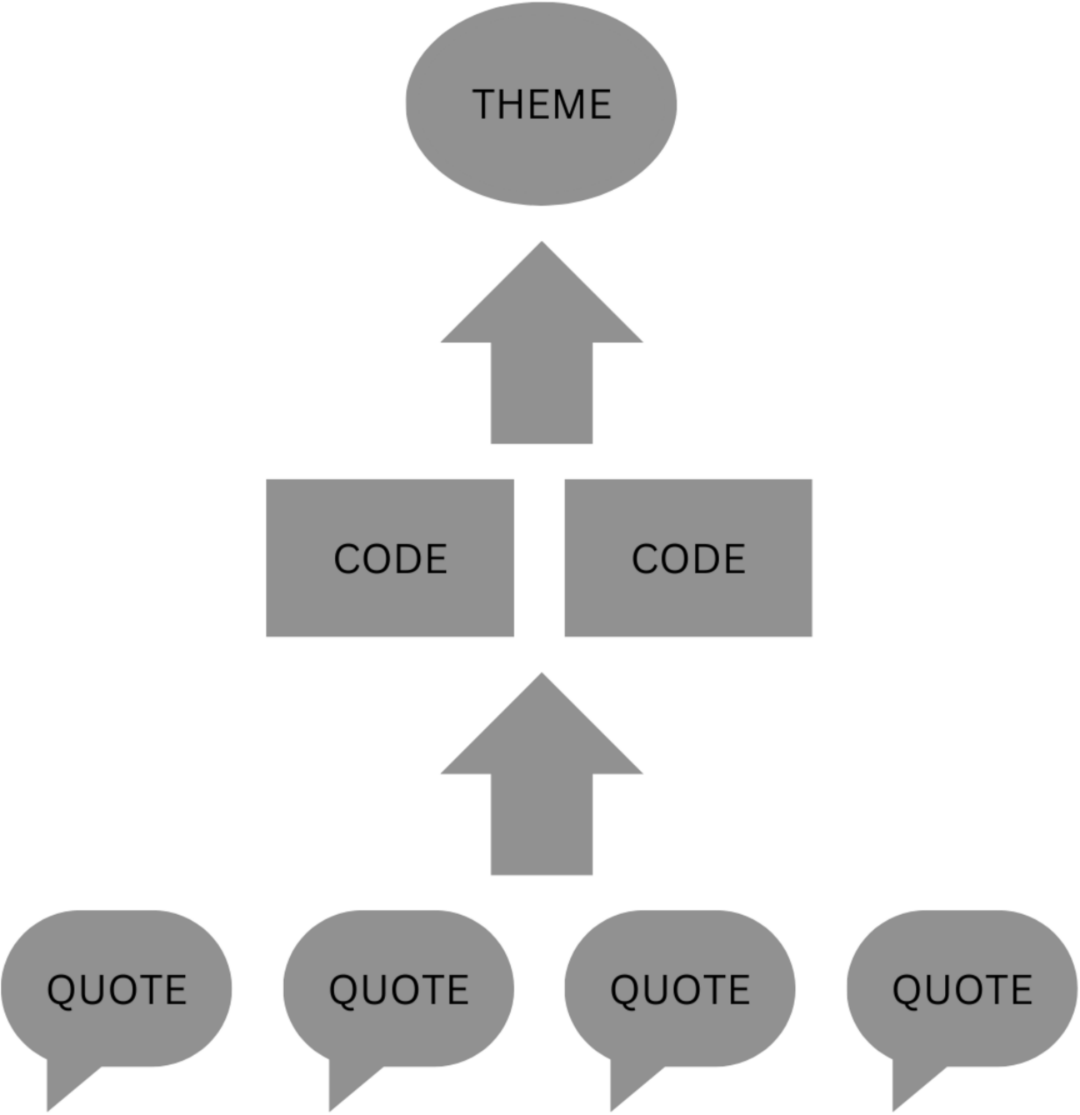
Thematic analysis process

Thematic analysis is a multi-stage process used to identify and examine patterns within qualitative data [12]. Analysts began by familiarizing themselves with the transcripts [11, 12]. Then transcripts were re-read to better understand participants’ meaning [11, 12]. During this process, the analysts applied meaningful descriptions to relevant sections of text, known as codes [11, 12]. These codes were then systematically applied across all transcripts, and finally codes were organized into overarching themes to describe patterns found throughout the data (Fig. 1) [11, 12]. For example, in the current study, the codes sleep loss; sleep quality; tired; nocturnal scratching; stress induced sleep loss; and sleep loss impacts on mood, were categorized into the overarching theme: atopic dermatitis severity negatively affects sleep quality and quantity for both mothers and infants. Constructs are considered saturated when no new constructs or explanations are identified in subsequent interviews, and a consensus has been reached between analysts on all overarching themes [11, 12].

This study was approved by the University of Manitoba Health Research Ethics Board HS24294 (H2020:426).

## Results

### Quantitative results

#### Maternal and infant characteristics

Overall, 32 mothers with an infant diagnosed with AD were included in this study. On average, mothers were 30.1 ± 4.2 years old, and 15/32 (46.9%) identified as White. Most (22/32; 68.8%) mothers had a university degree and 18/31 (58.1%) reported they were on some type of leave from their job. With consideration to their infants, the mean age was 8.3 ± 4.6 months, and a slight majority (19/32; 59.4%) were male (Table 2). The distribution of AD severity in the infants is shown in Table 2.

**Table 2:** Maternal and infant characteristics (*N*=32)

Variable	%	n	Mean	SD
**Maternal characteristics**				
Age			30.1	4.2
Number of adults in the home			2.3	0.9
Seasonality				
Winter	70%	21		
Spring	13.3%	4		
Fall	16.7%	5		
Ethnicity^a^				
White	46.9%	15		
Indigenous	28.1%	9		
Other visible minorities	25%	8		
University/college completed	68.8%	22		
Net annual household income (CAD)				
≤$50,000	31.3%	10		
$50,001 -$100,000	34.4%	11		
>$100,000	31.3%	10		
**Infant characteristics**				
Age (months)			8.3	4.6
Sex				
Female	40.6%	13		
Male	59.4%	19		
Allergic comorbidities^b^				
Food allergy	25%	8		
Asthma/wheeze	3.1%	1		
**AD treatment**				
Currently using moisturizers (*n*=30)	96.7%	29		
History of medication use	56.3%	18		
Other medicines used	12.5%	4		
**AD severity**				
Mild	56.3%	18		
Moderate	37.5%	12		
Severe	6.3%	2		

*Abbreviations* AD, atopic dermatitis; CAD, Canadian dollars; SD, standard deviation

^a^ Indigenous: First Nations, Métis. Other visible minorities: African, East Asian, South Asian, Other

^b^ Not mutually exclusive

#### Maternal and infant sleep outcomes

For the primary outcome, 6/14 (42.9%) of the mothers of infants with moderate/severe AD reported nocturnal awakening due to their infants’ AD, compared to 1/18 (5.6%) of the mothers of infants with mild AD. All together, 7/32 (21.9%) of mothers reported that their infant’s AD, regardless of severity, kept them awake at night, which was most commonly attributed to itching (85.7%), followed by worry (57.1%) (Table 3). Only 2/32 (6.3%) mothers reported that they “almost always” experienced difficulty sleeping even when they had the chance to, over the past month; whereas, 10/32 (31.3%) reported they did “not at all” experience difficulty sleeping when they had the chance to, over the past month. With consideration to infants, 4/30 (13.3%) were reported by their mothers to be awake at night due to AD, all four were affected by moderate/severe AD. Roughly 7% (2/30) of the infants received medication to help them sleep, with one infant having mild AD and the other having moderate/severe AD. Half (6/12) of mothers of infants with moderate/severe AD reported their infants suffered from a higher degree of sleeplessness (≥ 5 on a scale of 0–10) over the last 48 h, whereas none of the mothers of infants with mild AD 0/18 (0%) rated their infant’s sleeplessness as ≥ 5 over the last 48 h.

**Table 3:** Maternal sleep outcomes (*N* = 32)

Maternal sleep outcomes	Frequency (n)	Percent (%)
Infant’s AD causes maternal nocturnal wakening		
Yes	7	21.9
No	25	78.1
Causes of nocturnal wakening^a^ (*n* = 7)		
Itching	6	85.7
Need to treat	1	14.3
Stress	3	42.9
Worry	4	57.1
Other	0	0
Number of causes of maternal wakening (*n* = 7)		
1	2	28.6
> 1	5	71.4
Maternal report of difficulty sleeping over the past month, even when sleep was possible		
Yes	22	68.8
No	10	31.3
Maternal report of repeated memories, dreams or nightmares, over the past month		
Yes	10	31.3
No	22	68.8

*Abbreviations* AD, atopic dermatitis

^a^ Not mutually exclusive

### Associations between infant AD severity and maternal sleep

The relationship between AD severity and maternal sleep was analyzed using binary logistic regression, to consider potential confounders. The crude model resulted in a significant difference in nocturnal wakening if the infant had moderate/severe AD compared to mild AD (OR = 12.8, 95% CI 1.3–124.4, *p* = 0.03). In the partially adjusted model (controlled for infant age, maternal ethnicity, and maternal education) there was also a significant difference in adjusted odds ratio (aOR) (aOR = 90.4, 95% CI 2.0–4140.3, *p* = 0.02).

For the other primary outcomes, there was no significant difference in difficulty sleeping over the past month, even when sleep was possible, which was reported by 10/14 (71.4%) mothers of infants with moderate/severe AD compared to 12/18 (66.7%) mothers of infants with mild AD. For repeated memories, dreams or nightmares, over the past month, this was reported by 6/14 (42.9%) of mothers of infants with moderate/severe AD, compared to 4/18 (22.2%) of the mothers with infants with mild AD. However, these differences were not statistically significant in either the unadjusted or the adjusted model (Table 4). The fully adjusted model is found in Additional file 2.

**Table 4:** Associations between infant AD severity and maternal sleep outcomes, identified using logistic regression (*N* = 32)

Infant’s AD causes maternal nocturnal wakening					
		Unadjusted model	SE	Adjusted model^a^	SE
**Independent variables**	% of severity group endorsing dependent variable	OR (95% CI)		OR (95% CI)	
Mild AD	5.6	Ref	Ref	Ref	Ref
Moderate/severe AD	42.9	12.8 (1.3-124.4)*	14.8	90.4 (2.0-4140.3)**	176.4
**Maternal report of difficulty sleeping over the past month, even when sleep was possible**					
Mild AD	66.7	Ref		Ref	
Moderate/severe AD	71.4	1.3 (0.3–5.7)	1.0	1.1 (0.2–5.8)	0.9
**Maternal report of repeated memories, dream or nightmares, over the past month**					
Mild AD	22.2	Ref		Ref	
Moderate/severe AD	42.9	2.6 (0.6–12.2)	2.0	3.1 (0.5–17.6)	2.7

*Abbreviations* AD, atopic dermatitis; CI, confidence interval; SE, standard error; OR, odds ratio, Ref = reference category

^a^ Variables included in the adjusted model: Infant age, Maternal education, Maternal ethnicity

*p = 0.028, **p = 0.021

### Qualitative results

A total of 10 semi-structured interviews were completed with mothers of infants with AD averaging 30 min (range 17–52 min). Interviewees were 29.2 ± 4.96 years old, on average. The majority were married (90%) and had one child in the home (1.4 ± 0.5). Infants were 8.5 ± 4.93 months old and the majority were male (80%). Most infants had moderate AD (PO-SCORAD 34.53 ± 15.86) (Table 5). Based on the data from these interviews, we identified one theme: Atopic dermatitis severity negatively affects sleep quality and quantity for both mothers and infants.

**Table 5:** Qualitative participant demographics (*N*=10)

Variable	%	n	Mean	SD
**Maternal characteristics**				
Number of adults in the home			2.3	1.06
Seasonality				
Winter	90%	9		
Spring	10%	1		
Ethnicity^a^				
White	66.67%	6		
Indigenous	11.11%	1		
Other visible minorities	22.22%	2		
University/college completed	70%	7		
Annual net household income (CAD)				
≤$50,000	33.33%	3		
$50,001-$100,000	11.11%	1		
>$100,000	55.56%	5		
**Infant characteristics**				
Allergic comorbidities				
Food allergy	30%	3		
Severity of AD				
Mild	30%	3		
Moderate	60%	6		
Severe	10%	1		

*Abbreviations* AD, atopic dermatitis; CAD, Canadian dollars; SD, standard deviation

^a^ Indigenous: First Nations, Métis. Other visible minorities: African, East Asian, South Asian, Other

### Atopic dermatitis severity negatively affects sleep quality and quantity for both mothers and infants

Most mothers reported that AD impaired both their, and their infant’s sleep, mainly due to nighttime awakening subsequent to scratching and infant upset.

It has been rough because he was losing sleep so we were losing sleep, he has been waking up at night … starting to [scratch] and cry. – Participant 9.

If he is up, I am up. So definitely, he prevented us from sleeping … yes it was frustrating, but I mean you can’t control it at that point and you feel bad for him. – Participant 7.

For mothers, the stress of treating an infant with AD was also perceived to negatively affect sleep quality and quantity. In combination with the infant waking from discomfort, this resulted in perceived impacts on both the mothers’ and infants’ moods.

I feel like both of us were just miserable. – Participant 5.

Mothers described how when their infants’ AD worsened, such as during an exacerbation, both their infants’ and their own sleep quality and quantity would decrease.

He was probably getting up closer to 4 times when the eczema was really bad and once we got it under control it was dropping back down to 2, like you definitely see an increase in the amount of time he wakes up when his eczema breakouts are bad – Participant 7.

However, mothers described how, with consistent treatment, there could be improvements to both the infant’s AD and sleep quality. These positive changes did not just benefit infants, however, as some mothers also reported improved mood and stress following improvements in their child’s condition.

## Discussion

In this mixed methods study of mother-infant dyads, we quantitatively identified a significant association between AD severity and maternal sleep: mothers of infants with moderate/severe AD had greater negative sleep outcomes, including a statistically significant difference in nocturnal wakening, compared to mothers of infants with mild AD. In contrast, maternal difficulties sleeping even when possible and reports of repeated memories, dreams, or nightmares did not significantly differ across severity strata. Four infants, all of whom had moderate/severe AD, were affected in their sleep due to their AD. Qualitatively, we identified one theme “Atopic dermatitis severity negatively affects sleep quality and quantity for both mothers and infants’’. This confirmed and further explained the relationship between AD severity, the stress associated with treatment, and how exacerbation of the child’s AD can all impact maternal and infant sleep quality and quantity. In the following paragraphs, we have integrated the findings, and situate them within the broader literature.

Consistent with previous studies, our findings demonstrate a link between AD severity and poorer maternal sleep quality and quantity [5–7]. This was primarily experienced in the form of nighttime awakening, difficulty falling asleep, and reduced number of hours spent sleeping, which led to feelings of insufficient sleep and fatigue [6]. We found all of these to be associated with infant itching, the need to treat AD, stress, and/or worry. Interestingly, more mothers [4] than infants [3] reported being affected in their sleep due to their infants AD. Given that participants reported AD-related worries as a common cause of their sleep disturbances, this discrepancy between mothers and infants in their sleep outcomes might be related to stress/worry about their infant’s disease [13].

Findings from the quantitative measures suggest that sleep outcomes are worse among infants with moderate/severe AD relative to milder cases. This was reinforced in qualitative interviews as mothers described how when their infant’s AD worsened, their infant’s and their own sleep was negatively impacted. Taken collectively, the quantitative and qualitative results support that AD severity is associated with more negative maternal and infant sleep outcomes, with infant itchiness being the greatest factor.

Our findings not only corroborate previous studies by demonstrating a positive relationship between AD severity and negative sleep outcomes [5–7], but also extend previous research by solidifying findings in the infantile population, an area with minimal study to date. While the relationship between AD and infant sleep has received little study, it is nevertheless an important area of inquiry as sleep plays a significant role in infant development. Findings from the current study, which suggest greater AD severity is associated with poorer infantile sleep outcomes, provides some cause for concern as poor infant sleep has been found to predict impairments in infant mood, and can be associated with future developmental delays including behavioural issues and attention-regulation problems [14].

Not only can low sleep quality impact development, but it also has been shown to promote systemic inflammation [15]. Interestingly, some research has pointed towards a link between systemic inflammation and AD as well [16, 17]. While the relationship between systemic inflammation and AD is not totally understood, it is possible that sleep may have a bidirectional relationship with AD where AD contributes to poorer sleep and poorer sleep worsens AD [18]. Consistent with this reasoning, a study by Chang and colleagues, found that melatonin improved both sleep and disease severity in children with AD; however, it was not clear whether the reduction in disease severity stemmed from the improved sleep or the anti-inflammatory properties of melatonin [19]. While more research is needed to better understand the relationship between sleep and AD, initial findings suggest that promoting early interventions for those affected by AD may be important to help avoid a cascade of poor sleep and worsened AD outcomes.

Promoting improved sleep outcomes in infants with AD is also important for the sake of the parents. Research suggests maternal exhaustion as a result of infantile AD can result in poor parental outcomes including a reduction in bedtime emotional availability [20]. Parental practices at a young age can have lasting effects on a child potentially putting them at risk for adverse outcomes [20, 21]. Not only can exhaustion lead to poor parenting, which can have negative lifelong effects, but it can also lead to poor treatment adherence [6]. Ensuring treatment adherence is important as our findings suggest a relationship between consistent treatment and reduction in AD severity, leading to improved sleep outcomes for both mothers and infants.

We acknowledge the limitations of this study, most notably an underrepresentation of severe cases of AD. While unfortunate, the underrepresentation of severe cases likely reflects the young age of the sample as research suggests that the prevalence of severe cases increases with age [8]. However, because only two infants with severe AD were included in the quantitative sample and one in the qualitative sample, it is difficult to determine whether the findings from the current study generalize to infants with more severe manifestations of the condition. It should also be mentioned that the sample size was relatively small. We were originally hoping for a larger sample, however, our recruitment efforts were impacted by COVID-19-related restrictions limiting the number of patients receiving treatment and available for recruitment in-person at allergy and dermatology clinics. The low number of participants also restricted our ability to perform a fully adjusted regression model, due to few observations for several variables. Therefore, only a partially adjusted regression model was performed. Also, the low number of infants with nighttime wakening due to their AD, limited our ability to perform a regression analysis between infant sleep and infant AD severity. Furthermore, although the overarching study collected data from both maternal-infant dyads with and without AD, there was no sleep data collected from controls to provide a comparison. Future studies should attempt to recruit a larger sample, with more diversity in case severity, and a large control group to provide a comparison for sleep data.

Previous research has evaluated sleep quality and quantity in relation to AD, however, the literature is still quite limited and comprised primarily of quantitative studies. Our study utilized a mixed-methods approach to allow us to provide further insight into the lived experiences of mothers of infant’s with AD, something that has rarely been explored in relation to sleep [22].

In conclusion, the present study provides valuable information which not only confirms the association between AD severity and negative sleep outcomes but also extends this research into the infant population. In the current study, we were able to evaluate the perceived causes of maternal nocturnal wakening as well as assess the relationship between negative sleep outcomes for mothers and infants and how these might be greater in more severe cases of AD or during exacerbations. Finally, we propose that with adherence to topical treatment regimes, AD severity can be reduced, which can in turn lead to improvements in maternal and infant sleep quality and quantity.

### Electronic supplementary material

Below is the link to the electronic supplementary material.

Supplementary Material 1

Supplementary Material 2

## Data Availability

Owing to the highly personal nature of qualitative data, requests for data will be carefully vetted by a minimum of three authors. Requests for the quantitative data will be reviewed by the principal investigator.

## Abbreviations

AD: Atopic dermatitis
aOR: Adjusted odds ratio
CAD: Canadian dollars
CI: Confidence interval
DAG: Directed acyclic graph
OR: Odds ratio
PASS: Perinatal Anxiety Screening Scale
PO-SCORAD: Patient-Oriented Scoring of Atopic Dermatitis
Ref: Reference category
SD: Standard deviation
SE: Standard error
SES: Socioeconomic status
