# Supplementary material for: Infantile atopic dermatitis – increasing severity predicts negative impacts on maternal and infant sleep: a mixed methods study
Source: Allergy Asthma Clin Immunol. 2024 Mar 22;20:21. doi: 10.1186/s13223-024-00883-x (PMC10960393; doi:10.1186/s13223-024-00883-x)
Supplement: Supplementary file 1 — Supplementary Material 1 [file 13223_2024_883_MOESM1_ESM.pdf]

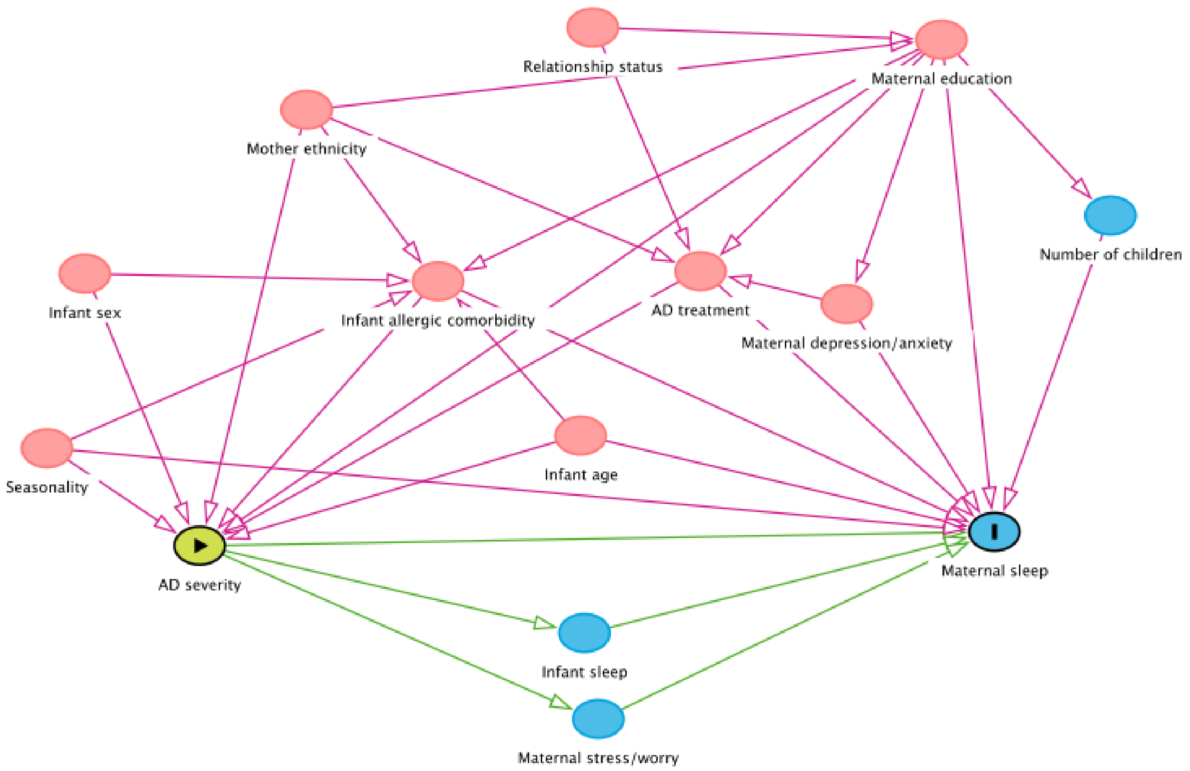

Panel a

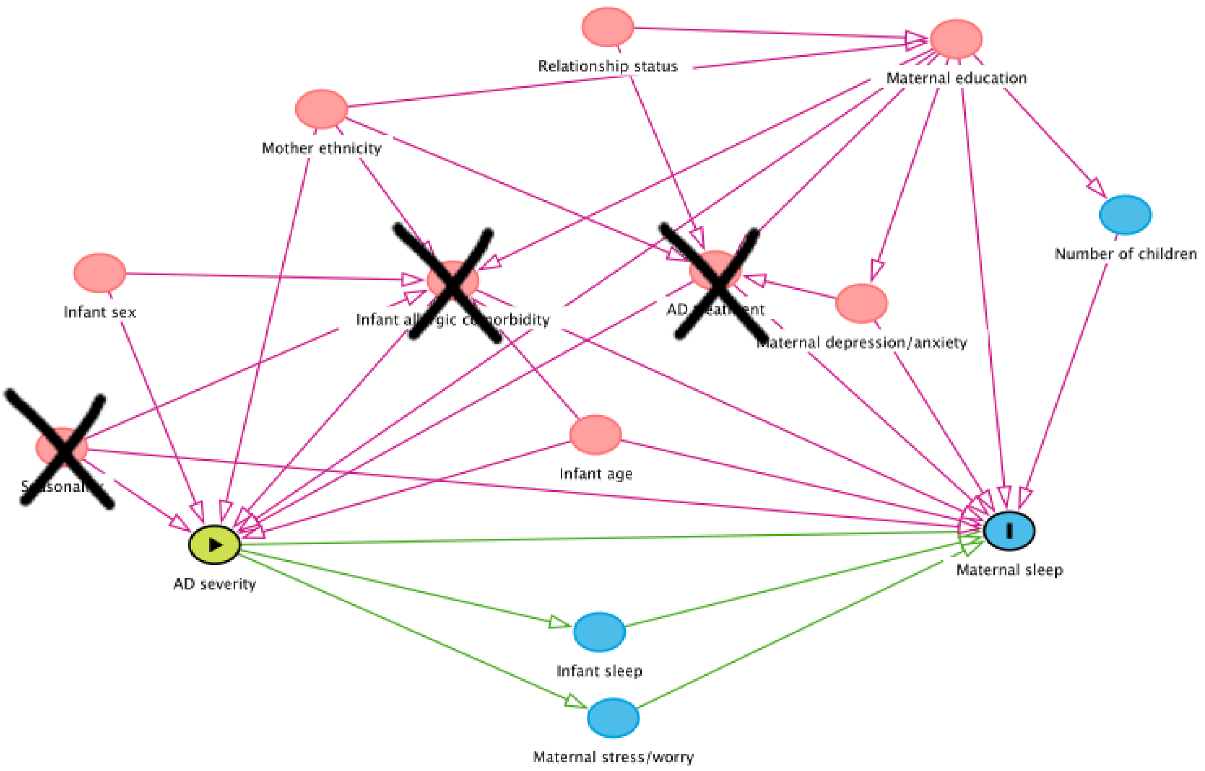

Panel b

eFigure 1. Directed acyclic graph (DAG) showing the associations between (a) possible confounders, exposure, and primary outcome, and (b) the confounders that were ultimately included in the model. Due to a low number of observations for infant allergic comorbidities (n=9) and few participants in each category for seasonality (winter =21, spring=4, fall=5), these variables were excluded from the model. AD treatment was excluded due to collinearity.

Abbreviations: AD, atopic dermatitis
AD severity is the exposure variable and maternal sleep is the outcome. Red circles indicate the ancestor of exposure and outcome (e.g., potential confounders), and blue indicates the ancestors of outcome (mediators). Green arrows indicate causal pathways and red arrows indicate biased pathways. Infant allergic comorbidities include asthma, rhinitis/hay fever, and food allergies.
