# Supplementary material for: Infantile atopic dermatitis – increasing severity predicts negative impacts on maternal and infant sleep: a mixed methods study
Source: Allergy Asthma Clin Immunol. 2024 Mar 22;20:21. doi: 10.1186/s13223-024-00883-x (PMC10960393; doi:10.1186/s13223-024-00883-x)
Supplement: Supplementary file 2 — Supplementary Material 2 [file 13223_2024_883_MOESM2_ESM.pdf]

eTable 1. Logistic regression of associations between maternal sleep outcomes and infant AD severity. Including the fully adjusted model without seasonality since when included, the regression model was not estimable.

Independent variables	n	N	%	Unadjusted model OR (95% CI)	Fully adjusted model[a] OR (95% CI)
**Infant's AD causes maternal nocturnal wakening (N=32)**					
Mild AD	1	18	5.6	ref	ref
Moderate/severe AD	6	14	42.9	12.8 (1.3-124.4)*	71.0 (0.8-6461.3)**
**Maternal report of difficulty sleeping over the past month, even when sleep was possible**					
Mild AD	12	18	66.7	ref	ref
Moderate/severe AD	10	14	71.4	1.3 (0.3-5.7)	1.8 (0.3-11.8)
**Maternal report of repeated memories, dreams, or nightmares, over the past month**					
Mild AD	4	18	22.2	ref	ref
Moderate/severe AD	6	14	42.9	2.6 (0.6-12.2)	9.0 (0.8-106.0)

Abbreviations: AD, atopic dermatitis; CI, confidence interval; OR, odds ratio; ref, reference category
[a] Variables included in Model 1: Infant age, Infant AD treatment, Infant allergic comorbidities, Maternal education, Maternal ethnicity.
*p=0.028, **p=0.064
